# Successful Mechanical Thrombectomy of a Middle Cerebral Artery Occlusion 14 Hours after Stroke Onset

**DOI:** 10.1155/2017/9289218

**Published:** 2017-10-04

**Authors:** Anelia Dietmann, Kety Wha-Vei Hsieh, Andrea M. Humm, Claudio L. Bassetti, Urs Fischer, Jan Gralla

**Affiliations:** ^1^Department of Neurology, Inselspital, University Hospital Bern, University of Bern, Bern, Switzerland; ^2^Department of Neuroradiology, Inselspital, University Hospital Bern, University of Bern, Bern, Switzerland; ^3^Division of Neurology, Department of Internal Medicine, HFR Fribourg-Cantonal Hospital, Fribourg, Switzerland

## Abstract

A 54-year-old patient presented with mild right-sided weakness of hand and face with a National Institutes of Health Stroke Scale (NIHSS) of 2 and occlusion of the left middle cerebral artery (MCA) in the M1 segment with a large perfusion deficit on computed tomography (CT). Due to mild neurological deficits no IVT was performed. Nine hours after symptom onset the patient gradually deteriorated with a NIHSS fluctuating between 9 and 15. MRI showed a persistent occlusion of the MCA with a large diffusion-perfusion mismatch. Immediate endovascular thrombectomy was performed 14 hours after symptom onset with complete recanalization and complete clinical recovery. Although mechanical thrombectomy is generally considered an effective alternative strategy up to 8 hours after stroke onset, selected patients with a large diffusion/perfusion mismatch and small infarct cores may benefit from an expanded therapeutic window.

## 1. Background

For two decades intravenous thrombolysis (IVT) has been the most important therapy for patients with an acute ischemic stroke [1]. However, eligibility of IVT is limited due to a narrow time window of 4.5 hours after symptom onset with a rapidly decreasing efficacy and various contraindications such as comorbidities, oral anticoagulation, or recent surgery. Furthermore, recanalization rates and outcome in patients with large clots and proximal vessel occlusion are poor after IVT [2].

Recently, data from several randomized clinical trials demonstrated a clear benefit of endovascular treatment in patients with acute ischemic stroke caused by a proximal vessel occlusion in the anterior circulation [3]. Selection of patients based on imaging—especially targeting for diffusion/perfusion mismatch and excluding patients with a large infarct core—has been shown to successfully avoid futile interventions in all these trials [4].

Currently the time window for favorable outcome after endovascular treatment is not well defined. Endovascular treatment with stent retriever thrombectomy is recommended up to 8 hours after symptom onset [5].

## 2. Case Presentation

**Figure 1:**
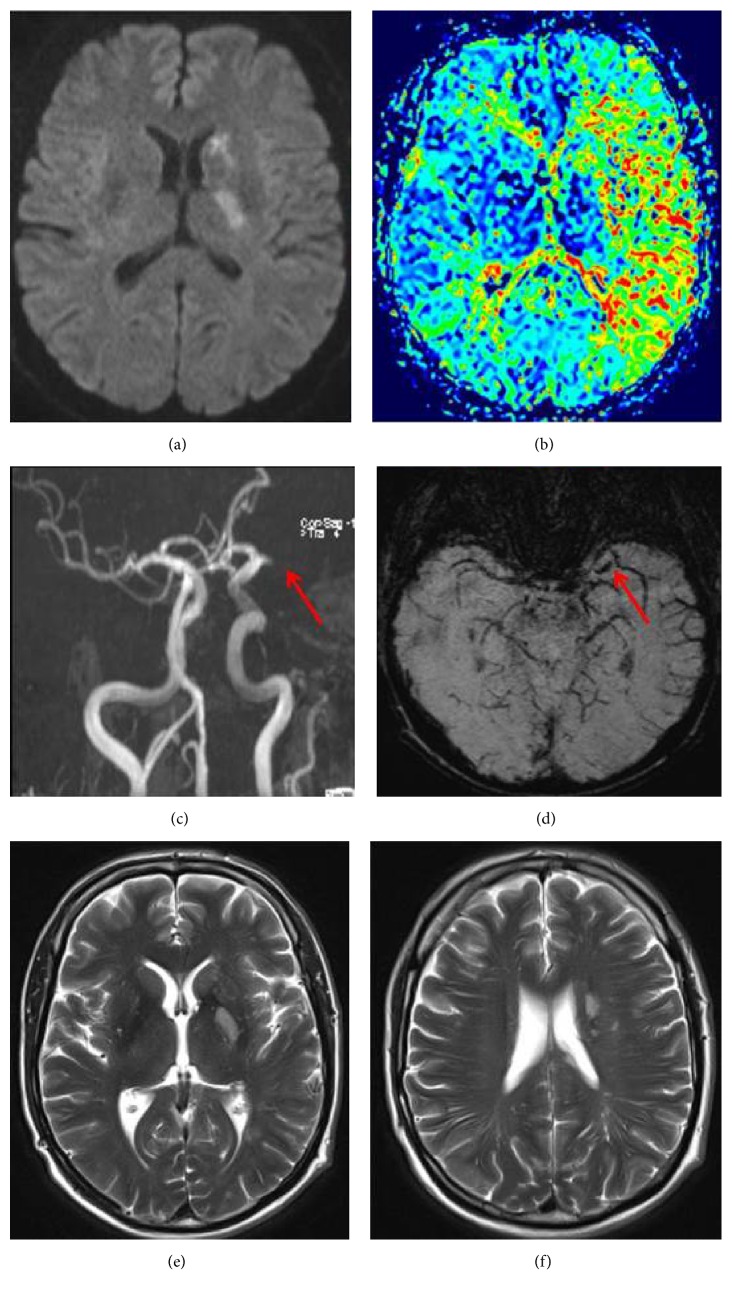
Cerebral magnetic resonance imaging 13 hours after stroke onset with a small diffusion lesion in the left basal ganglia and corona radiata with a complete perfusion deficit of the left MCA territory (a, b). Persistent occlusion of the MCA on magnetic resonance angiography ((c), red arrow) and susceptibility weighted imaging ((d), red arrow). T2 weighted imaging 24 hours after the intervention showing a small left sided basal ganglia infarction (e, f).

A 54-year old, right-handed previously healthy male patient presented with an acute right sided weakness and facial palsy to a peripheral hospital (NIHSS on admission: 2). Computed tomography (CT) angiography 4 hours and 20 minutes after symptom onset showed an occlusion of the left MCA in the M1 segment with a large perfusion deficit in the complete MCA territory. Since clinical symptoms were very mild the treating physicians decided against IVT and the patient received best medical treatment, that is, 250 mg acetylsalicylic acid intravenously, Atorvastatin 40 mg per os, and enoxaparin 40 mg s.c. He was transferred to the local stroke unit for further monitoring. Within nine hours after symptom onset the patient gradually deteriorated, without any obvious cause (e.g., seizure, hypotension, and fever). After transferal to our stroke center he presented with severe aphasia and sensorimotor right-sided hemiparesis with an NIHSS fluctuating between 9 and 15. Immediately after admission to our hospital (i.e., 13 hours after stroke onset), the patient underwent a cerebral magnetic resonance imaging (MRI) (Figure 1) showing a small diffusion lesion in the left basal ganglia and corona radiata with a complete perfusion deficit of the left MCA territory and a persistent occlusion of the MCA on magnetic resonance angiography (MRA). The subsequent cerebral distraction angiography (DSA) confirmed the complete occlusion of left MCA in the M1 segment. After consent of the family the interventional neuroradiologists performed mechanical thrombectomy in conscious sedation. Complete recanalization was achieved with the Solitaire retriever in one attempt 14 hours after stroke onset. Angiography at the end of the intervention showed a complete reperfusion of the former occluded left MCA territory (TICI classification 3).

NIHSS immediately after the intervention was 4, and on the following day 2 with a mild weakness of the right arm. MRI 24 hours after intervention showed small left sided basal ganglia infarction (Figure 1). The patient was retransferred to the regional hospital 36 hours after intervention. One week after symptom onset the patient reported to have completely recovered.

## 3. Discussion

Endovascular treatment in acute stroke patients with occlusion of large vessels in the anterior circulation improves functional outcome [6]. Recanalization is associated with better outcome and recanalization rates with mechanical techniques have been shown to be superior to pharmacological treatment only [6]. Patients with mild or rapidly improving symptoms, but proximal vessel occlusions have a poor outcome when left untreated [6]. In an earlier study especially proximal vessel occlusion and NIHSS ≥ 10 were predictors of poor outcome patients with stroke with mild or rapidly improving symptoms [7]. Therefore, rapid identification and treatment of these patients are of major importance. In cases of large vessel occlusion and low NIHSS with decision against treatment in the acute phase, transferal to a stroke center with endovascular treatment facilities for further monitoring is highly recommended.

However, the time window for favorable outcome after endovascular treatment is not well defined. Only very recently first results of the DAWN study—presented on the 3rd European Stroke Organisation Conference in Prague and not jet fully published—showed that removal of a clot by endovascular thrombectomy within up to 24 hours after onset of signs and symptoms reduced the disability in selected stroke patients, such as wake-up strokes [8]. As demonstrated by Flint et al. and confirmed by Jovin et al. earlier, endovascular treatment with stent retriever thrombectomy within 8 hours after symptom onset is safe and reduces the severity of poststroke disability [5, 9]. Whether mechanical thrombectomy is beneficial in selected patients after 8 hours remains unclear. Multimodal MRI imaging techniques have been shown to better identify patients that are likely to benefit from endovascular reperfusion therapy. Particularly patients with a significant perfusion/diffusion mismatch on MRI have substantial salvageable brain tissue that can be recovered from ischemia after reperfusion by endovascular treatment, showing better long-term clinical outcome [4]. Although mechanical thrombus extraction is generally an alternative strategy for acute revascularization up to 8 hours after stroke onset [5, 9], selected patients may benefit from an expanded therapeutic window. Therefore, further studies—and final results from the DAWN trial—are needed to better define time windows and multimodal imaging protocols for save and efficient endovascular treatment in patients with acute ischemic stroke.
